# Critical appraisal on mitochondrial dysfunction in Alzheimer’s disease

**DOI:** 10.1002/agm2.12217

**Published:** 2022-07-25

**Authors:** Faizan Ahmad, Punya Sachdeva

**Affiliations:** ^1^ Department of Medical Elementology and Toxicology Jamia Hamdard University Delhi India; ^2^ Amity Institute of Neuropsychology and Neurosciences Amity University Noida Uttar Pradesh India

**Keywords:** Alzheimer's disease, mitochondrial dysfunction, neurodegenerative disease

## Abstract

It is widely recognized that Alzheimer's disease (AD) is a common type of progressive neurodegenerative disorder that results in cognitive impairment over time. Approximately 152 million cases of AD are predicted to be reported by 2050. Amyloid plaques and tau proteins are two major hallmarks of AD which can be seen under electron microscope. Mitochondria plays a vital role in the pathogenesis of AD and mitochondria disruption leads to mitochondrial DNA (mtDNA) dysfunction, alteration of mitochondria dependent Ca2+ homeostasis, copper dysfunction, immune cell dysfunction, etc. In this review, we try to cover all the mechanisms related with mitochondrial dysfunction and mitochondrial pathogenesis that may help us to better understand AD as well as open a new era for therapeutic target of AD and treat this progressive disease.

## Graphical abstract

Amyloid plaques deposit on neurons in case of Alzheimer's Disease. Mitochondria play a vital role in pathogenesis of Alzheimer's Disease. Mitochondrial dysfunction can lead to formation of mPTP, alteration of complexes 1,3 and 4, deficits in bioenergetics with impaired mitochondrial biogenesis.

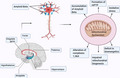

## 1 INTRODUCTION

**FIGURE 1:**
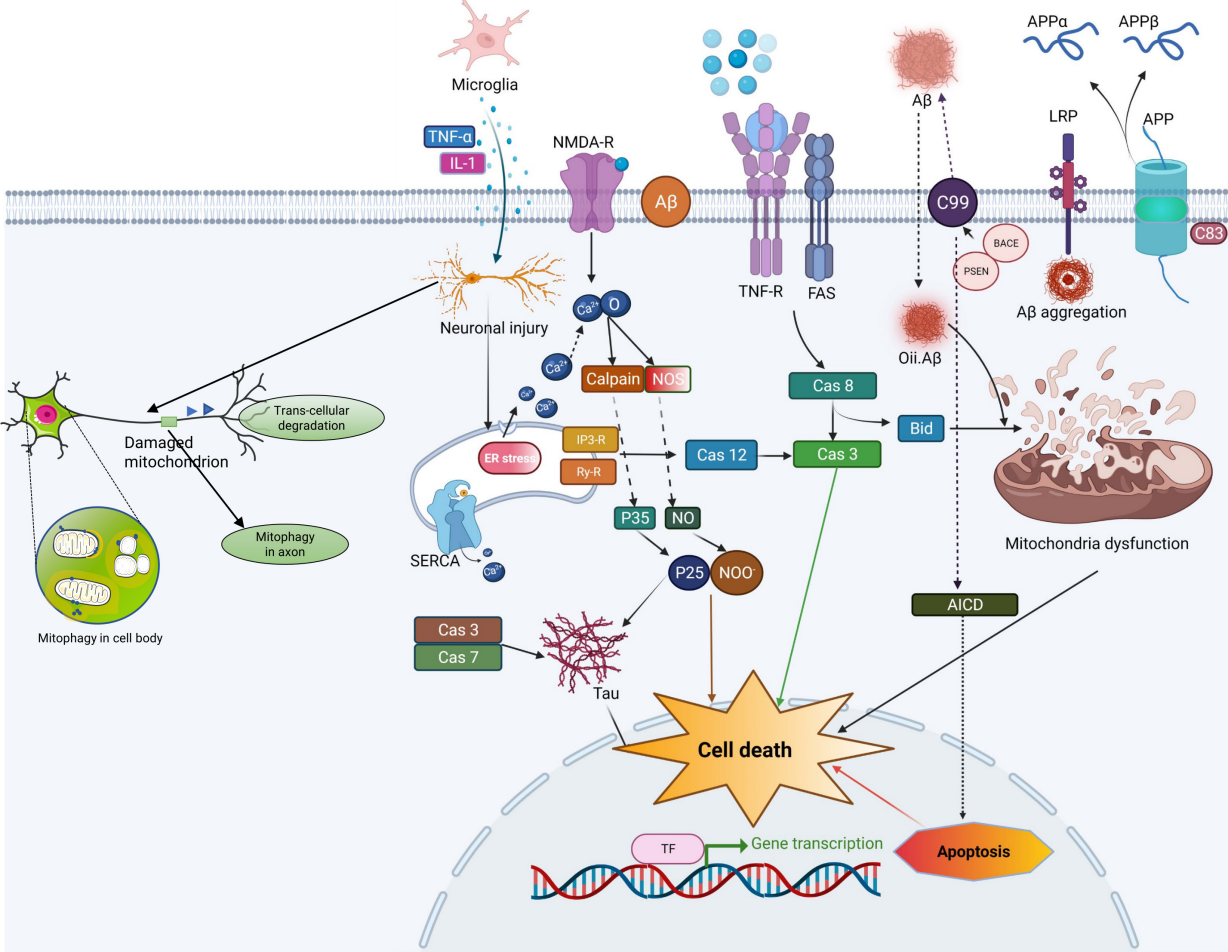
Cell death in AD is mainly due to amyloid deposits and there is activation of caspase 3, which is one of the most important factors in plaque formation and neuronal death. AD, Alzheimer's disease.

Currently, there is no holistic treatment for managing Alzheimer's disease (AD), a degenerative brain illness. A loss of cognitive abilities characterizes AD. When it progresses slowly but steadily, it becomes increasingly complex and lethal. Several repercussions result from the disease's course, including a steady deterioration of cognitive and memory function over time. Patients with AD experience short‐term memory loss, making it difficult to carry out their daily responsibilities efficiently and successfully. In AD, along with these limitations, problem‐solving abilities might develop.^1^, ^2^, ^3^ According to the Global Alzheimer's Report 2010, AD is the most common cause of dementia, accounting for more than 60% of all cases of the condition worldwide. Almost from its beginning, AD has had a substantial influence on the international economy, with the global economy incurring a cost of more than $800 billion in a single year alone.^2^, ^3^ It is essential to investigate the causes, methods of diagnosis, and potential treatments associated with this illness to resolve this public health issue. Given the relevance of this disorder in public health, it is vital to explore the causes, methods of diagnosis, and potential treatments connected with it. AD is characterized pathologically by the formation of intracellular neurofibrillary tangles (NFTs), senile plaques, and the extracellular accumulation of Aβ in the brain, which contains abnormal tau protein types. According to the findings of several investigations, these aggregates and their antecedents are accountable for neuronal dysfunction, which results in cognitive and memory impairment. A striking finding is that in both the human and murine models of AD, disruption of mitochondrial activities caused by tau, amyloid‐beta, or both pathogenic aggregates has been identified much earlier than the formation of NFTs and amyloid plaques, which are considered to be the disease's defining characteristics.^4^, ^5^ Various mechanisms, such as mitochondrial energy transfer, vesicle transport, antioxidant defenses, and synaptic connection, are responsible for the brain's energy transport. Patients who have AD may suffer from cognitive impairment due to these flaws in their brain.^3^, ^4^, ^5^ Hardy and Higgins were the first to propose the amyloid cascade hypothesis, first published in 1992 and has since gained widespread acceptance. A recent study has demonstrated that the amyloid cascade hypothesis, which has been around for decades, is a more critical component in the development of the disease than the persistence of the disease. The use of peptides may be necessary in some cases.^6^, ^7^, ^8^ However, it is not conducive to AD development, as evidenced by its variety of manifestations. Despite further investigation in AD animal models, there was no evidence of a link between the disintegration of senile plaques of amyloid fibrils and neuronal cell death. This suggests that oligomers may be the primary cytotoxic agents in this condition.^6^, ^7^, ^8^ Mitochondrial dysfunction has been observed in the synapses of patients with AD regularly, and this has been linked to the disease's origin and progression. This hypothesis posits that the drop‐in mitochondrial activity that happens with aging is responsible for various physiological changes in the nervous system, including memory loss. To reimburse for and mend these alterations, the cell makes an effort. Still, after reaching a certain level, such payback becomes impossible, resulting in the formation of distinctive symptoms of the disease. Therefore, mitochondrial genetics and environmental factors have a significant role in determining the clinical diagnosis of AD. When these and several other conformations are coupled, mitochondrial dysfunction becomes the focus of attention, as it is the primary actor in the pathogenesis of AD.^8^, ^9^ The most apparent symptom of AD is mitochondrial dysfunction, which can be seen in the brain. Mitochondria are the organelles that are responsible for the production of ATP. Mitochondria must perform all critical biological tasks, including apoptosis (through caspase‐independent and caspase‐dependent mechanisms), the generation of reactive oxygen species, and the maintenance of calcium homeostasis in cells.^10^ Consequently, mitochondrial dysfunction has been associated with the pathophysiology of AD, leading to the establishment of the mitochondrial cascade hypothesis. According to a recent study, amyloid promotes the upregulation of VDAC1, resulting in the establishment of the 1‐methyl‐4‐phenyl‐1,2,3,6‐tetrahydropyridine (mPTP) barrier in the brains of APP transgenic mice. According to the findings of this investigation, tau that has been hyperphosphorylated integrates with VDAC1, which indicates the development of another mitochondrial malfunctioning procedure. The cortical material from a patient with AD postmortem has been connected to amyloid discovered in mitochondria and mAPP transgenic mice, each of which has a different component of mPTP. According to the results, this appeared to promote reactive oxygen species (ROS) generation and neuronal cell death in the mouse model.^11^, ^12^, ^13^, ^14^ After taking into account all of the studies, a conclusion was reached regarding how mitochondrial‐encoded gene expression increased in AD; there are numerous mechanisms by which Aβ associates with AD mitochondria and how mitochondrial dysfunction may aid in AD‐related transformation, illustrating the need for further field investigation in this area of study.^15^, ^16^ The consequences of mitochondrial dysfunction in AD are depicted in Figure 1.

## 2 MITOCHONDRIAL DNA's FUNCTION IN AD

As a result of somatic mutations in mitochondrial DNA (mtDNA), AD is genetic. The genetic makeup of a person's mtDNA increases their chances of contracting the disease. When determining the presence of related genes from nuclear and mitochondrial genomes in a given individual, the mitochondrial genome is the most important factor. Mitochondrial dysfunction is the primary mechanism through which AD impairs cognition, and neurons in this condition are particularly susceptible to this malfunction. According to some research, a 4997‐bp deletion, referred to as “the common deletion,” develops due to normal aging and has been associated with AD development in some cases.^17^, ^18^, ^19^, ^20^, ^21^, ^22^ According to some experts, somatic mtDNA deletions eliminate AD in humans because they strongly increase in places with high metabolic activity. Researchers detected more mtDNA alterations associated with AD than in age‐matched controls. High deletion rates are identified regularly in the early stages of the development of AD cortical neurons.^22^ When it comes to patients with AD over 80 years of age, the common deletion rate is consistently lower than younger people. The age‐matched control cortex of persons with younger brains shows the polar opposite trend, with lower overall deletion rates and higher rate acceleration as they get older, respectively, compared with those who are younger or with younger brains.^21^, ^22^, ^23^ According to scientists (mtDNA), mutations in mtDNA are caused by oxidative damage caused by AD. As a result, mutations in the mtDNA regulatory area are becoming increasingly widespread in the frontal cortex of persons who have AD, according to recent research.^22^, ^23^, ^24^ In response to a drop in mtDNA transcription and replication rates, mutations in the regulatory region become more common (mtDNA). Following this, using next‐generation sequencing (NGS), it was discovered that patients with AD had higher levels of mtDNA point mutations in the hippocampus than those without the condition. After investigating AD inheritance patterns, including mtDNA inheritance as a potential risk factor for AD, the authors conclude that point mutations in AD are most likely caused by errors in mtDNA replication or oxidative destruction.^21^, ^22^, ^23^, ^24^ According to research findings, individuals who have a family history of AD are more prone than the general population to suffer from cerebral hypometabolism, which may increase their chances of developing AD over time. As a result of the fact that maternal DNA is predominantly passed from mother to child, the bias of AD against maternal inheritance is dependent on the direction in which AD obtained the impact from mtDNA at the time of discovery. According to the findings, different conformations of mtDNA have been revealed through hybrid studies of mtDNA associated with mitochondrial abnormalities in AD.^24^ In hybrid formation, exogenous mtDNA is introduced into cells that do not have mtDNA, leading them to divide and eventually reproduce. Platelet‐derived exogenous mtDNA can also be obtained from the patient's blood cells, enabling the creation of hybrids that contain mtDNA from the patient with AD. As a result, when hybrids are placed on a stable nuclear basis, they can accurately mimic the mitochondrial activity associated with AD.^25^ Genetically engineered AD cybrids can reproduce a wide range of characteristics linked with AD. Early AD cybrid research discal, which imitated the mitochondria of people with AD, had a COX activity impairment, confirmed by further experiments. AD cybrid COX deficiencies are present, and it is mtDNA responsible for the mitochondrial abnormalities related to the disease. Since then, it has been established that mtDNA deletions in the Alzheimer's hippocampus result in COX deficits and that neurons with deficient COX proliferate in the Alzheimer's hippocampus, according to the findings of additional research. A significant increase in mtDNA deletions was identified in the COX‐deficient AD neurons, showing that these deletions are leading to COX scarcity in these neurons. According to their findings, researchers have discovered that mitochondrial malfunction in AD hybrids may play a role in improving neuropathology in patients with AD. Hybrids of AD and Parkinson's disease have amyloid changes similar to those seen in AD and an increase in their vulnerability to amyloid beta‐fragments.^25^, ^26^ Compared with control groups, the amyloid‐beta production of AD hybrids is markedly more than that of typical AD cells.^23^, ^24^ It has been shown that elevated amyloid‐beta levels are related to increased cytochrome C release and caspase‐3 activity, indicating that activation of the cell death pathway may contribute to the elevation of amyloid‐beta levels in the circulation. According to the findings, amyloid‐beta were statistically significant improvements in several parameters of AD hybrids treated with amyloid‐beta, including metal, cytochrome C release, and caspase three activity. These improvements were observed across all of the hybrids. The amyloidosis feature of the mitochondria is responsible for improving the cells' resistance to amyloid‐beta evolution and the death of cells. This is accomplished by regulating the formation of amyloid‐beta in the mitochondria.^26^, ^27^

## 3 FISSION, FUSION, AND FUNCTION: MITOCHONDRIAL DYNAMICS IN NEURONAL CELLS OF AD

Depending on the demands of the surrounding environment, mitochondria are constantly proliferating and fusing within cells, resulting in a steady flow of energy. Neurons place a high strain on the mitochondrial function of their cells. A cell's ability to operate effectively necessitates the expenditure of vast quantities of energy. Microorganisms, specifically mitochondria, are responsible for a large proportion (90%) of all energy given to a cell through the cell's metabolism supplied to the cell, as well as for a wide range of ATP‐dependent neuronal functions, including vesicle release, synaptic transmission, ion channel and receptor‐related actions, and neurotransmitter recycling and re‐consuming, among others. Fission and fusion are the two most important mechanisms by which the mitochondria maintain synchronization with the cells' energy demands. Furthermore, these tactics allow mtDNA and metabolites to spread during fusion processes while retaining the fraction of damaged mitochondria in the cell during fission at a lower level of deterioration during fusion and fission. Specific guanosine triphosphatase (GTPase) enzyme activity is regulated mainly by both mechanisms.^28^, ^29^ When required, DLP‐1 (also known as DNM1L) is thought to be recruited to the outer mitochondrial membrane, whereas Fis1 is a tiny protein that is thought to be recruited to the outer mitochondrial membrane when required. One of two mitochondrial processes is activated when a single mitochondrial division occurs: fission and fusion. In addition to being identified as a DLP‐1 receptor, Fis1 has also been connected to the recruitment of DLP‐1 to the cell. However, for the intracellular material to fuse, the inner membrane must also fuse, and it is at this point that OPA1 enters the picture to help. To interfere in the fusing of the inner mitochondrial membrane (IMM), OPA1 interacts with the intermembrane space. It requires Mfn1, but not Mfn2, to do so. Thus, Mfn1 is required, but not necessary, for OPA1 to aid with the fusing of the IMM microtubules. With regard to mitochondrial function, various elements must be considered. Considering the position of the mitochondria within a cell and how many mitochondria are present in that cell at any given time are both crucial elements to consider.^30^, ^31^ Mitochondria can move about within cells because of the tracks left by the cytoskeleton, which allows them to do so. According to the findings, whereas the mitochondrial matrix and the intracellular Ca^2+^ concentration are critical in controlling axonal mitochondrial motility, neuronal activity is responsible for most of the increase in the number of moving mitochondria in the axon. It is believed that the aberrant fission and fusion of mitochondria, along with the formation of elongated and short circular mitochondria, results in the morphological changes in the distribution of mitochondria inside the cell. Aside from this, the cytoskeleton is critical in maintaining cell integrity, and transformation in this way, such as the neurodegeneration found in AD, can have disastrous effects on the integrity of the cells involved.^32^

## 4 ALTERATION OF MITOCHONDRIA‐DEPENDENT CA^2+^ HOMEOSTASIS IN AD

According to research, calcium imbalance has been identified as a condition primarily associated with the dysfunction of subcellular organelles, such as mitochondria in AD. Multiple studies have connected improper calcium regulation to mitochondrial dysfunction, in addition to the other factors that have been mentioned previously. Even though Na^+^/Ca^2+^ exchanger (NCX) is one of the essential regulators of intracellular calcium levels, its involvement in the pathophysiology of the nervous system is still not completely understood.^33^, ^34^, ^35^, ^36^ This is particularly true in the case of AD, in which NCX is emerging as a transporter that may be involved in the pathophysiology of the central nervous system, according to recent research. As per the results from the recent study conducted by John et al; 2020 have indicated that NCX is also a critical regulator of cellular metabolism, working at both the plasma membrane and mitochondrial levels of the cell.^34^ Energy metabolism and intracellular calcium levels have been hypothesized in recent years. It has been suggested that deficiencies in energy and calcium signaling may be the earliest reversible concerns in AD. Reduced NCLX activity during a mouse AD model resulted in more than mitochondrial calcium and oxidative stress, culminating in amyloid and tau pathology and neuronal death.^34^, ^35^, ^36^, ^37^ Furthermore, it has been discovered that neuropathies impact the quantities of calcium in the body's tissues, which is a good thing.^35^, ^36^, ^37^ Several studies have linked mitochondrial failure to neuropathy, most notably in the case of diabetic neuropathy. They have discovered that it is associated with altered cellular calcium homeostasis and aberrations in mitochondrial Ca^2+^ storage. The presence of diabetes causes greater intracellular Ca^2+^ concentrations in sensory neurons, notably those of the lumbar dorsal root ganglia, which is related to higher mitochondrial Ca^2+^. It is believed that this state results in depolarization of the mitochondrial membrane, which can aid in the development of oxidative stress, the production of ROS, and the development of mitochondrial functional abnormalities, all of which can lead to calcium dyshomeostasis throughout the body. On the other hand, acetylcholine and antibodies against cholinergic receptors successfully protect neurons from amyloid‐induced cell death; nevertheless, they are both ineffectual when protecting neurons from amyloid‐induced Ca^2+^ dysregulation.^38^, ^39^, ^40^, ^41^, ^42^ Calcium‐dependent molecules, such as calmodulin, and the proteins that bind to them are thought to play a vital role in AD, and they have been proposed as potential indicators of the condition. Researchers discovered that tau‐induced NCLX depletion resulted in faster mitochondrial depolarization than when the tau protein was present when neurons were subjected to (pathological) recurrent Ca^2+^ stimulations. As demonstrated by this finding, cell death caused by Ca^2+^ is more common in neurons than in other cells. As a result of the FTD‐associated 10 + 16 mutation in MAPT, patients' induced pluripotent stem cell (iPSC)‐derived neurons were more sensitive to physiological and pathological Ca^2+^ stimulation than controls. This resulted in greater mPTP susceptibility.^38^ According to the researchers, their findings give the first convincing biological proof that mCa^2+^ efflux loss promotes the development of AD and that enhancing pathogenic mCa^2+^ clearance could be a promising therapy option for delaying the course of AD.^42^

## 5 ENERGY METABOLISM DEFICIENT IN AD

The mitochondria, which are the energy‐producing powerhouses of the cell, are essential for the cell's ability to utilize energy. Their eradication can significantly impact the cell's ability to do so. AD in the brain is further evidenced by the absence of the tricarboxylic acid cycle's two rate‐limiting enzymes (alpha‐ketoglutarate dehydrogenase complex [KGDHC] and pyruvate dehydrogenase complex [PDHC]), both required for glucose metabolism in the brain. This suggests that the brain's glucose metabolism has been impaired.^43^, ^44^ There has indeed been a substantial amount of research into metabolic irregularities in AD, and one of the most well‐known anomalies in AD is the brain's ability to process information at a slower rate than usual. In addition, there is solid evidence that oxidative stress is higher in patients with AD when compared to glucose stress, which is of particular importance. Following some evidence of functional handicap by neuropsychological testing of brain atrophy by brain imaging is more effective.^43^, ^44^ All of the just causes of behavioral and neurological abnormalities in AD (eg, hypoxia, hypoxia‐induced hypoxia, and hypoglycemia) can be found in other metabolic illnesses comparable to AD independently. The researchers discovered that activating canonical Wnt signaling decreases symptoms in AD animal models, mainly through boosting glycolytic enzymes and improving glucose metabolism, as reported in the journal *Nature Neuroscience*.^43^ The increased synthesis of glycolytic ATP may be beneficial in treating energy deprivation. The hydrotropic impact of ATP may aid in the solubility and clearance of toxic aggregates, joint in many neurological illnesses, including AD. Inflammatory neurodegenerative diseases are caused by mitochondrial malfunction, which decreases the amount of energy available to neurons and speeds up the onset of the disease. As a result, it is prudent to include treatment of energy metabolism issues in the treatment strategy.^43^, ^44^, ^45^ According to the experts, creating a neurodegenerative disease treatment that incorporates the adjustment of energy metabolism would be highly beneficial in the long term. A genetically maintained adaptation mechanism, which allows animals to manage with low oxygen levels by reducing their reliance on mitochondrial oxidative metabolism, permits them to live longer lives due to this adaptation mechanism. Animals can activate this adaptive mechanism when they are exposed to hypoxia. This metabolic shift may prove beneficial in treating neurodegenerative illnesses such as AD.^44^, ^45^, ^46^

## 6 DYSFUNCTION OF COPPER AND MITOCHONDRIA IN AD

AD is characterized by a breakdown in the body's ability to regulate the amount of copper.^47^ Under the conclusions of a research study carried out by professionals, patients with AD had higher copper (II) concentrations in their cerebrospinal fluid (CSF) than those without the disease. This study determined that the neurotoxicity of Cu (II), an artificially conditioned environment caused Aβ 1–40 on primary microglia derived from rats, and the results demonstrated that it was neurotoxic. When it was observed that Cu (II)‐A1‐40 complexes induced an increase in the formation of mitochondrial ROS in the V‐2 microglia cell line, it was considered a breakthrough at the time. In the BV‐2 cell line, N‐acetylcysteine (NAC) therapy led to a significant reduction in TNF‐alpha and nitric oxide production, which were considered positive outcomes.^48^, ^49^, ^50^ According to the findings of the study, when the Cu (II)‐A1‐40 complex was used as a model, there was no evidence of acute neurotoxicity in the presence of the Cu (II)‐A1‐40 complex in the conditioned channel from NAC‐treated primary rat microglia when the Cu (II)‐A1‐40 complex was used.^51^, ^52^ For the idea that copper is involved in the mitochondrial malfunction of microglia in AD to be supported by experimental evidence, additional research is required.

According to several studies, ionized metals have been implicated in regulating homeostatic brain function and the progression of disease processes, particularly those linked with AD.^53^ It is necessary to conduct additional research to understand better the association between mitochondrial failure in microglia and AD, which is one of the study's objectives. Researchers have discovered that in transgenic mice that express the AD model protein APP/PS1, which is seen in AD, the intracellular bioavailability of Cu2+ in complex Cu2+−containing compounds such as bis‐(thiosemicarbazone) rises. Several favorable effects were observed due to treatment, including a drop in amyloid trimer content, an increase in neuroprotective cellular signaling pathways activation, suppression of GSK3, and a decrease in tau protein phosphorylation. This study also demonstrated that cognitive processes in mice of this line treated with bis (thiosemicarbazone) were restored to normalcy, which was a shocking revelation at the time of publication.^47^ It has been demonstrated that creating a bond with amyloid results in the development of oligomers capable of piercing cell walls in the presence of Cu2+. Following an extended period of exposure to Cu, researchers revealed that rats suffered from spatial memory impairment, which was linked to mitochondrial damage in the hippocampal formation, as reported in the findings (hippocampus). As a result of the findings, persistent copper exposure, in particular, has been demonstrated to worsen the beta‐amyloid‐induced memory loss reported in rats, and this has been proven to be particularly true for rats.^49^ We hypothesize that chronically elevated cytosolic Ca2+ signaling in AD neurons can result in mitochondrial dysfunction. Based on our findings, we believe that drugs that suppress or stabilize neuronal calcium signaling and mitochondrial activity may have therapeutic potential in treating AD in the elderly population. Insufficient metal ion balances can contribute to the development of amyloid‐beta and tau disease due to the varied effects of metal ions on various cellular and subcellular functions, as mentioned previously.^50^, ^54^, ^55^ The destabilization of homeostasis can further worsen pre‐existing abnormalities in metal ion transit and deposition in the brain. As a result, scientists have come up with a hypothesis that modifying the body's mental homeostasis by the supplementation or chelation of metal ions may ameliorate AD's pathologies, therefore opening up new research routes for treating the condition. Researchers have already reported a copper shortage in the brains of those who have AD, and our data complement this discovery.^50^, ^51^, ^52^ Furthermore, they suggest that medications aimed at safely and successfully increasing copper levels in the brain could constitute a novel experimental‐therapeutic approach to the disorder.^50^

## 7 P2X7R AND INSTABILITY OF MITOCHONDRIA IN AD

It is still unclear what the P2X7 receptor (P2X7R) does in non‐immune cells, such as neurons, even though it is well‐known for its role as a “death receptor” in the immune cell.^56^ P2X7R antagonists, which have a high bioavailability and excellent penetration into the central nervous system while simultaneously having a low incidence of adverse effects, are being investigated as a potential treatment for neurodegenerative illnesses.^57^ The amyloid‐induced mitochondrial mortality in microglia depends on the P2X7 receptor for survival and proliferation, rather than the other way around (P2X7R). It has been demonstrated that the treatment of microglia with amyloid results in the activation of NLRP3, mitochondrial toxicity, and transcriptional activation of the nuclear factor NFdB, among other effects. Microglia derived from P2X7R−/− mice, and N13 cells with decreased P2X7R‐expression revealed a significant increase in mitochondrial potential compared to controls after being exposed to amyloid for 24 hours.^58^ The difference in mitochondrial potential between microglia isolated from P2X7R−/− mice and N13 cells with decreased P2X7R‐expression was still less but statistically significant in microglia isolated from N13 cells with decreased P2X7R‐expression. The difference in mitochondrial potential between microglia isolated from P2X7R−/− mice and N13 cells with decreased P2X7R‐expression was still less but statistically significant in microglia isolated from (N13R). As a result of the addition of A to N13 cells, a further rise in ROS production was seen, which was reliant on the presence of the P2X7R molecule.^58^ CytC is a protein that plays a function in transmitting amyloid‐induced mitochondrial damage throughout the body. Compared to cells treated with vehicle control, cells treated with amyloid demonstrated a significant increase in mitochondrial CytC‐release and cytosol accumulation.^56^ N13R cells were shown to have a 30% lower release of CytC than the other cell types, according to the findings. When cytokines are produced, apoptosis in microglia is promoted, contributing to the etiology of AD in human beings. In addition, the calcium channel blocker nimodipine effectively inhibits the microglial inflammatory response and rescues mitochondria from damage caused by amyloid. More in‐depth research is required to conclude that amyloid affects the membrane potential of mitochondria in microglia and that the ramifications are significant.^56^, ^57^, ^58^

## 8 AD AND MITOPHAGY DEFICIENCY

AD is associated with mitochondrial malfunction and chronic neuroinflammation, and several pathogenic processes, including neuronal death.^58^ It is common for a mitochondrial malfunction to occur during the dynamic mitochondrial cycles, which is a vital proponent of the mitophagy pathway and a significant contributor to the accumulation of waste products. Although their numbers are dropping, microtubule‐associated mitochondria (also known as mammalian mitochondria) may undergo fission and fusion and compress in size while their numbers increase.^59^, ^60^ Critical resources can combine and sustain mitochondria through fusion, mitochondria can expand through fission, and defective accumulation can be removed. A cytoplasmic network of microtubules, known as mitochondrial transport, allows mitochondria to move across the cell and interact with other organelles. Neuronal cells undergo biogenesis down long axons containing dendrites, but only after mitochondrial axonal transport is essential. Performing selective autophagy (also called mitophagy) in mitochondrial malfunction is critical for bringing about a close to the mitochondrial dynamic cycle and preventing further damage to the cell.^59^, ^60^ In addition to the presence of features, such as oxidative damage, cytochrome C leakage from the IMM, the release of calcium from the IMM, membrane potential loss, caspase activation, and mtDNA damage, mitochondrial malfunction is also characterized by the presence of features such as mtDNA damage.^60^, ^61^ These characteristics appear in the body before the commencement of selective autophagy occurs. It is the process by which damaged mitochondria are removed from the mitochondrial network, consequently increasing the proportion of normal mitochondria and biogenesis compared to the fraction of faulty mitochondria and oxidative stress in the mitochondrial network.^62^ The fact that the coding gene poly‐(ADP‐ribose) polymerase 1 (or PARP1) and the Sirtuin SIRT1 share a shared resource (NAD+) is also well known. As a result, the two genes are supposed to be in a competitive relationship for that resource shortly. As a result, biogenesis is restricted, and mtDNA damage occurs due to the aging process, exacerbating the already‐existing mitophagy dysfunction. According to recent research published in peer‐reviewed scientific literature, apoptosis is caused by the failure of mitophagy, and neuronal death is a component of the pathogenesis of AD. To maintain control over mitochondrial efficiency, one must first ensure that the mitochondria dynamics are balanced. As demonstrated by the development of defective mitochondria, which are occasionally structurally deficient and aesthetically deformed in AD cells, AD cells exhibit aberrant autophagy.^63^, ^64^ Decreased levels of the anti‐apoptotic proteins parkin and PINK1 in the cytoplasm may have led to the high levels of amyloid accumulated in the cytoplasm, which may have resulted in a decrease in the rapidly increasing number of autophagosomes that target damaged mitochondria. Researchers discovered that the lysosomes in AD neurons were malfunctioning and were also smaller in size than usual. Liposomes that are defective in injured neurons transport proteolytic substrates to the proteasome, which is responsible for down damaged neurons. Several mitophagy initiating proteins, including ULK1 and TBK1, have been discovered in human AD postmortem tissue and AD iPSC derived cortical neurons, suggesting that they may be a possible underlying cause for the accumulation of damaged mitochondria.^63^ In light of these findings, it is plausible to postulate that if impaired mitophagy is a contributing factor to the etiology of AD, restoring mitophagy may be able to partially reverse and even prevent the evolution of the AD phenotype in some instances. Using *C. elegans* models of AD, including the 3xTgAD animal model of AD and the APP/PS1 mouse model of AD, it was discovered that mitophagy inducers, such as the small molecules urolithin A (UA) and action (A), are present at lower levels in the brains of these AD animal models.^63^ The aging process has been demonstrated to play a significant part in the development and pathophysiology of AD, among other things, by impairing the function of synapses, the autophagic system, and the mitophagy system, all of which have been mentioned previously. The need for additional investigation cannot be overemphasized to better understand microglial activation and mitochondrial damage in neurons, especially at synapses. Despite discovering numerous links between Aβ and tau and interactions between Aβ and tau and mitochondrial proteins and mitophagy components, the specific mechanisms and sequence of events that contribute to the start of AD have not yet been identified. Because several different beneficial forms of mitophagy have been implicated in the development of AD, addressing mitochondrial dysfunction with chemicals that target mitophagy may aid in the development of a potential therapeutic and preventative strategy for people suffering from the condition.^60^, ^61^, ^64^ It has been demonstrated that mitophagy is not functioning properly well in the hippocampus of people with AD.^65^ In nematode and rat models of AD, both mitophagy‐inducing drugs improved the survival and form and function of neurons that use glutamate and choline. They also stopped amyloid‐ and tau pathologies, and the animals' memories improved. These findings suggest a common way that memory loss happens across the different AD models. This common way is caused by a problem with mitophagy.^66^ Intervention strategies that boost mitochondrial health and stimulate mitophagy may indeed avert the neurodegenerative process in AD.^67^

## 9 FUNCTION FOR MITOCHONDRIAL MPTP IN AD

The mPTP may be activated if an increase in oxidative stress accompanies the increase in intra‐mitochondrial Ca^2+^. This will result in cell death and mitochondrial depolarization, both of which are potentially lethal. The findings of several studies have led to the hypothesis that amyloid could be transported directly to the mitochondrial matrix by binding to cyclophilin D, which is a significant regulator of mPTP opening found in the mitochondrial matrix. The findings of several studies support this hypothesis.^68^, ^69^ Alternatively, it has been demonstrated that anion‐selective channel‐1 has a conductivity dependent on the voltage applied. Using confocal microscopy, a recent study proved that amyloid could be absorbed into the cell, confirming the concept that A can be internalized into cells. Intriguingly, it was discovered that the mitochondria in the DS astrocytes were condensed, a finding proven to be compatible with mitochondrial fragmentation and associated with the synthesis of mitochondrial PTP. Therefore, it is possible that Aβ, by inhibiting complex IV and complex V and binding to A‐binding alcohol dehydrogenase, will promote mitochondrial malfunction and the generation of ROS in the cell. Further research has indicated that tau is involved in complex I deregulation and is a transcription factor.^65^, ^66^, ^67^ Research is now being conducted on MitoTEMPO as a potential therapy for AD. A study was carried out to determine the toxicity of amyloid‐beta in primary cultures of mouse neurons. It was found that, despite a significant drop in neuronal lipid oxidation, the bioenergetics of mitochondria was preserved, as evidenced by the maintenance of mitochondrial membrane potential, cytochrome C activity, and ATP generation study. Because CypD is the only mPTP component that has been identified to date, it is possible that inhibiting CypD will prove to be a feasible therapy option in the not‐too‐distant future.^68^, ^69^, ^70^

## 10 CONCLUSION AND FUTURE DIRECTIONS

AD is a complex disorder with no known cause that has not yet been discovered and currently has no effective treatment. It is currently unclear what role the mitochondrial genome plays in AD. There are several obstacles to overcome before getting a thorough grasp of the mitochondrial genome AD connection. However, due to limitations of inaccessible data and research approaches, it is still possible to analyze the role of mitochondrial genetics in the development of AD and other neurodegenerative illnesses, despite evidence to the contrary. In light of the discovery that mitochondrial dysfunction plays a substantial role in pathogenesis, more significant inquiry into how mitochondria become dysfunctional in the first place will be required. The “mitochondrial cascade theory” proposes that the accumulation of somatic mitochondrial DNA mutations with continuous aging influences the activity of neurons in the brain, and it is based on this idea. However, the data gathered to support this idea have been inconsistent, necessitating additional inquiry.

## AUTHOR CONTRIBUTIONS

*Manuscript writing and drawing figures*: Ahmad. *Manuscript reviewing and editing*: Sachdeva.

## FUNDING INFORMATION

No funding was received for this study.

## CONFLICT OF INTEREST

The authors declare they have no conflict of interest.

## CONSENT FOR PUBLICATION

All authors have given consent for publication.
